# A Comparative Study of Molybdenum Carbonyl and Oxomolybdenum Derivatives Bearing 1,2,3-Triazole or 1,2,4-Triazole in Catalytic Olefin Epoxidation

**DOI:** 10.3390/molecules24010105

**Published:** 2018-12-28

**Authors:** Lucie S. Nogueira, Patrícia Neves, Ana C. Gomes, Tatiana A. Amarante, Filipe A. Almeida Paz, Anabela A. Valente, Isabel S. Gonçalves, Martyn Pillinger

**Affiliations:** Department of Chemistry, CICECO—Aveiro Institute of Materials, University of Aveiro, Campus Universitário de Santiago, 3810-193 Aveiro, Portugal; lucienogueira@ua.pt (L.S.N.); pneves@ua.pt (P.N.); agomes1@ua.pt (A.C.G.); tatiana.amarante@ua.pt (T.A.A.); filipe.paz@ua.pt (F.A.A.P.); igoncalves@ua.pt (I.S.G.)

**Keywords:** molybdenum, carbonyl complexes, triazole, oxidative decarbonylation, epoxidation

## Abstract

The molybdenum(0)-carbonyl-triazole complexes [Mo(CO)_3_(L)_3_] [L = 1,2,3-triazole (1,2,3-trz) or 1,2,4-triazole (1,2,4-trz)] have been prepared and examined as precursors to molybdenum(VI) oxide catalysts for the epoxidation of *cis*-cyclooctene. Reaction of the carbonyl complexes with the oxidant *tert*-butyl hydroperoxide (TBHP) (either separately or in situ) gives oxomolybdenum(VI) hybrid materials that are proposed to possess one-dimensional polymeric structures in which adjacent oxo-bridged dioxomolybdenum(VI) moieties are further linked by bidentate bridging triazole (trz) ligands. A pronounced ligand influence on catalytic performance was found and the best result (quantitative epoxide yield within 1 h at 70 °C) was obtained with the 1,2,3-triazole oxomolybdenum(VI) hybrid. Both molybdenum oxide-triazole compounds displayed superior catalytic performance in comparison with the known hybrid materials [MoO_3_(trz)_0.5_], which have different structures based on organic-inorganic perovskite-like layers. With aqueous H_2_O_2_ as the oxidant instead of TBHP, all compounds were completely soluble and active. A pronounced ligand influence on catalytic performance was only found for the hybrids [MoO_3_(trz)_0.5_], and only the 1,2,4-trz compound displayed reaction-induced self-precipitation behavior. An insight into the type of solution species that may be involved in the catalytic processes with these compounds was obtained by separately treating [MoO_3_(1,2,4-trz)_0.5_] with excess H_2_O_2_, which led to the crystallization of the complex (NH_4_)_1.8_(H_3_O)_0.2_[Mo_2_O_2_(*μ*_2_-O)(O_2_)_4_(1,2,4-trz)]·H_2_O. The single-crystal X-ray investigation of this complex reveals an oxo-bridged dinuclear structure with oxodiperoxo groups being further linked by a single triazole bridge.

## 1. Introduction

Molecular and polymeric molybdenum oxides that incorporate organic moieties as either charge-balancing counter-ions or ligands have proven to be excellent catalysts for several oxidation reactions in organic synthesis [1,2,3,4,5]. Some illustrative and significant examples of molybdenum(VI)-catalyzed reactions described in the last fifteen years include complexes of the type [Cp’MoO_2_X] (Cp’ = unsubstituted or substituted cyclopentadienyl, X = halide, alkyl, alkyl ester, *ansa*-bridged alkyl or cycloalkyl group, etc.) for the epoxidation of olefins with *tert*-butyl hydroperoxide (TBHP) [4,6], with activities that surpass that of the CH_3_ReO_3_/H_2_O_2_ system, enantioselective oxidation of olefins catalyzed by a chiral bishydroxamic acid complex [7], enantioselective sulfoxidation using a chiral bisguanidinium dinuclear oxodiperoxomolybdosulfate catalyst [8], amino triphenolate complexes as catalysts for sulfoxidation, epoxidation and haloperoxidation [9], a polyoxomolybdate-calix[4]arene hybrid as a heterogeneous catalyst for sulfoxidation [10], selective olefin epoxidation using nanoparticle-supported, magnetically recoverable complexes [11], and reaction-induced self-separating catalysts for the oxidation of olefins, sulfides and alcohols, based on octamolybdate salts [12] or a MoO_3_-triazole hybrid material [13].

A common feature of molybdenum(VI)-catalyzed reactions is that the starting complex acts as a precatalyst, being transformed under the reaction conditions to oxoperoxo-molybdenum(VI) complexes, which may be the actual catalysts responsible for activating the oxidant toward the oxidation reaction [14]. Experimental and computational studies showed this to be the case with the cyclopentadienyl complexes [Cp’MoO_2_X], which react with the oxidant ROOH to give species of the type [Cp’MoO(O_2_)X] [15,16,17,18,19]. An important discovery was that the tricarbonyl complexes [Cp’Mo(CO)_3_X] could be used as precatalysts rather than the dioxomolybdenum(VI) complexes [4]. In analogy with the Arco-Lyondell process for the epoxidation of propene, where the precursor Mo(CO)_6_ is oxidized in situ by TBHP to a dioxomolybdenum(VI) complex [20], the tricarbonyl complexes undergo rapid oxidative decarbonylation in the presence of excess TBHP to form complexes of the type [Cp’MoO_2_X], [Cp’MoO(O_2_)X], [(Cp’MoO_2_)_2_(*μ*-O)] and [(Cp’MoO(O_2_))_2_(*μ*-O)] [4,6,21]. This work inspired studies with other types of molybdenum carbonyl complexes and led to the isolation of a variety of mononuclear, dinuclear, polynuclear and polymeric molybdenum(VI) oxides. Examples include [CpMoO_2_(NHC)]BF_4_ from [CpMo(CO)_2_(NHC)(CH_3_CN)](BF_4_) (NHC = *N*-heterocyclic carbene) [22], tetranuclear [Mo_4_O_12_(pypz)_4_] from *cis*-[Mo(CO)_4_(pypz)] (pypz = 2-[3(5)-pyrazolyl]pyridine) [23], octanuclear [Mo_8_O_24_(di-*t*Bu-bipy)_4_] from *cis*-[Mo(CO)_4_(di-*t*Bu-bipy)] (di-*t*Bu-bipy = 4,4′-di-*tert*-butyl-2,2′-bipyridine) [24], polyoxomolybdate salts containing the anion [β-Mo_8_O_26_]^4−^ from *cis*-[Mo(CO)_4_(L)] (L = chiral 7-(1-pyrindanyl)amine ligand) [25], and the one-dimensional (1D) molybdenum oxide/bipyridine polymer [MoO_3_(2,2′-bipy)] from *cis*-[Mo(CO)_4_(2,2′-bipy)] (2,2′-bipy = 2,2′-bipyridine) [24].

In the present work, the molybdenum(0)-carbonyl-triazole complexes [Mo(CO)_3_(L)_3_] [L = 1,2,3-triazole (1,2,3-trz) or 1,2,4-triazole (1,2,4-trz)] have been prepared and examined as precursors to molybdenum(VI) oxide catalysts for olefin epoxidation using TBHP or H_2_O_2_ as oxidant. One motivation for this research was the finding mentioned above that the MoO_3_-triazole hybrid material [MoO_3_(1,2,4-trz)_0.5_] could be used as a self-separating catalyst for oxidation reactions with aq. H_2_O_2_ as oxidant [13]. In a later study, the reaction of MoO_3_ with H_2_O_2_ in the presence of 1,2,4-trz led to the isolation of the trinuclear oxoperoxo complex (1,2,4-Htrz)_2_[Mo_3_O_6_(O_2_)_4_(1,2,4-trz)_2_], which constituted the first case of a discrete Mo^VI^ complex bearing triazole ligands [26]. These studies point to the untapped potential of oxomolybdenum(VI)-triazole compounds in oxidation catalysis. A particular focus of the present work was to compare in parallel the chemistry and catalytic behavior of the two tricarbonyl complexes containing the isomeric ligands, and also the hybrid materials [MoO_3_(1,2,3-trz)_0.5_] and [MoO_3_(1,2,4-trz)_0.5_], which have isomorphous layered perovskite-like structures [13,27,28]. Transition-metal 1,2,3-triazole-based catalysts have attracted major interest [29,30] and the examination of isostructural catalysts bearing 1,2,3-trz or 1,2,4-trz ligands should be of great value in improving our understanding of ligand influences on catalytic performance.

## 2. Results and Discussion

### 2.1. Catalyst Preparation

**Scheme 1:**
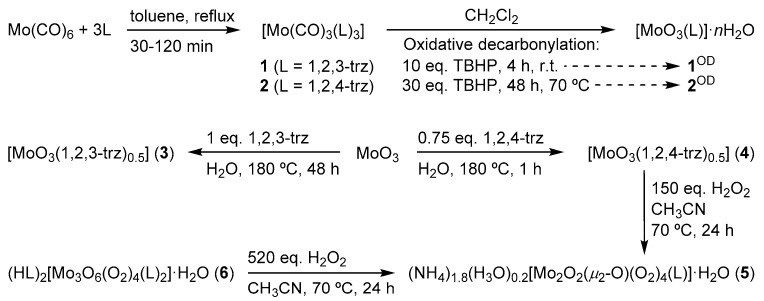
Preparation of **1**–**5**, **1**^OD^ and **2**^OD^.

The triazole tricarbonyl complexes [Mo(CO)_3_(1,2,3-trz)_3_] (**1**) and [Mo(CO)_3_(1,2,4-trz)_3_] (**2**) were obtained in very good to excellent yields by the direct reaction of Mo(CO)_6_ with three equivalents of the organic ligand in refluxing toluene (Scheme 1). Both complexes **1** and **2** display limited stability in the solid-state if stored cold in the dark and under inert atmosphere. Complex **2** decomposes within seconds upon exposure to air to give a black solid. Complex **1** is partially soluble in diethyl ether, and insoluble in pentane. Complex **2** is highly insoluble in polar and nonpolar solvents. The poor solubility of **2** is in line with the known characteristic of the unsubstituted 1,2,4-triazole ligand to produce microcrystalline/insoluble precipitates immediately with transition metal ions in solution [31].

**Figure 1:**
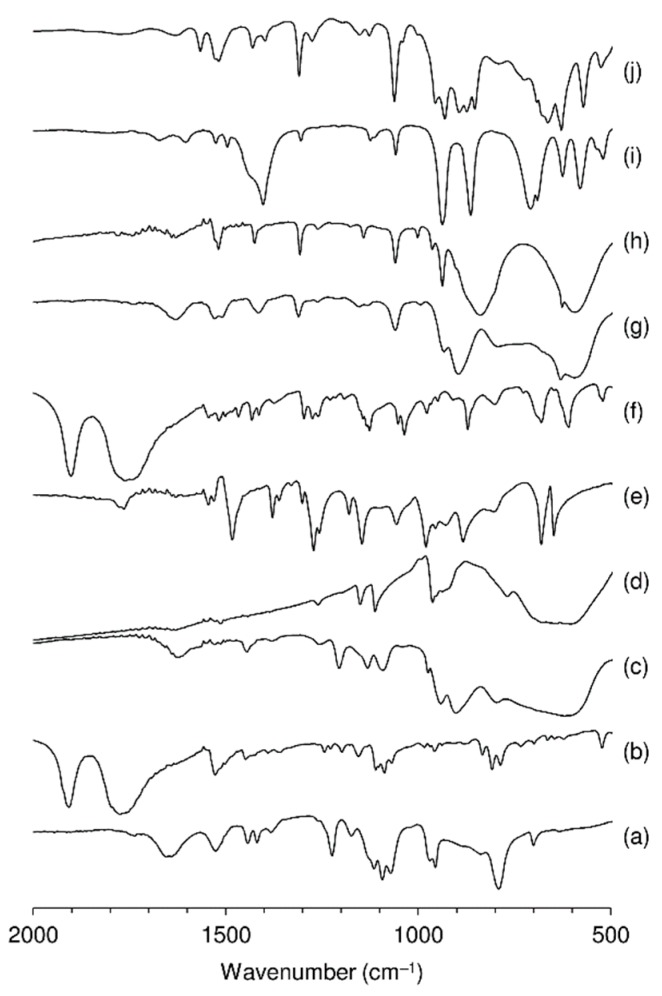
FT-IR spectra in the range of 500–2000 cm^−1^ of (**a**) 1,2,3-trz, (**b**) tricarbonyl complex **1**, (**c**) oxidized derivative **1**^OD^, (**d**) hybrid **3**, (**e**) 1,2,4-trz, (**f**) tricarbonyl complex **2**, (**g**) oxidized derivative **2**^OD^, (**h**) hybrid **4**, (**i**) dinuclear complex **5**, and (**j**) trinuclear complex **6** (from ref. [26]).

For **1** and **2** the carbonyl stretching region of the IR spectra contains two bands near 1905 cm^−1^ (A_1_ mode) and 1760 cm^−1^ (E mode), as expected for facial tricarbonyl complexes with approximate C_3v_ symmetry (Figure 1b,f) [32]. The lower-energy band is broadened and split, presumably due to low site symmetry in the solid-state [32]. These observations are in line with the general rule that octahedral [M(CO)_3_L_3_] complexes (L = planar N-donor heterocycle such as pyridine or azole) occur exclusively as the *fac* isomer [33,34]. The spectra for **1** and **2** are in agreement with that reported for *fac*-[Mo(CO)_3_(Hpz)_3_] (Hpz = pyrazole), which showed three strong absorptions at 1897, 1777 and 1730 cm^−1^ due to carbonyl stretching [34]. In Figure 1 the spectra of solid 1,2,3-trz and 1,2,4-trz are included for comparison. In the region containing the triazole ligand modes (500–1600 cm^−1^), the spectra of **1** and **2** resemble those of the free ligand; this is especially noticeable for **1** and 1,2,3-trz. 

**Figure 2:**
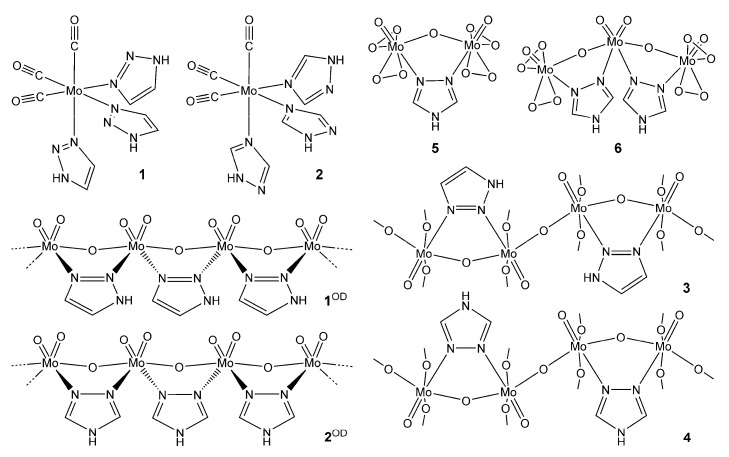
Schematic representations of the proposed structures for **1**, **2**, **1**^OD^ and **2**^OD^, and the known structures for **3**–**6**.

According to Haasnoot et al., a monodentate triazole coordination mode (rather than a bridging one) is indicated when the IR spectrum of a complex resembles the spectrum of solid triazole [35]. For 1,2,3-trz, N3 is a better donor than N2 [29], and therefore we may expect that in **1** the ligands will be coordinated through N3. For structurally characterized complexes containing only monodentate 1,2,4-trz, the coordination is always through N4 [36]. These coordination modes are represented in Figure 2.

Oxidative decarbonylation (OD) of **1** and **2** was carried out by the dropwise addition of 5–6 M TBHP (10 equiv.) in *n*-decane to a suspension of the tricarbonyl complex in CH_2_Cl_2_. The resultant suspensions were filtered to isolate the solids referred to as **1**^OD^ and **2**^OD^. For **1**, performing the reaction at ambient temperature for 4 h was sufficient to achieve complete decarbonylation (verified by the absence of CO stretching absorption bands in the FT-IR spectrum of the solid product). For the much less soluble complex **2**, a longer reaction time and higher temperature (70 °C, 48 h) were required to achieve complete decarbonylation. Microanalyses (CHN) and TGA data (Figure S1 in the Supporting Information) for **1**^OD^ were consistent with the composition [MoO_3_(C_2_H_3_N_3_)]·H_2_O.

The presence of a polymeric molybdenum oxide microstructure in **1**^OD^ and **2**^OD^ is supported by the FT-IR spectra which present several common features in the Mo‒O stretching region (500–1000 cm^−1^). The very broad band centered around 610 cm^−1^ for both compounds may be assigned to a *ν*_as_(Mo‒O‒Mo) vibration, while the strong bands near 900 and 940 cm^−1^ may be assigned to terminal *ν*(Mo=O) stretching modes (Figure 1c,g). The presence of a pair of *ν*(Mo=O) bands is indicative of *cis*-[MoO_2_]^2+^ structural units, with the lower-energy band being the asymmetric vibration and the higher-energy band being the symmetric vibration [37]. For both **1**^OD^ and **2**^OD^ the pattern of bands in the Mo‒O stretching region closely resembles that observed for the 1D hybrid molybdenum oxide/bipyridine materials [MoO_3_(2,2′-bipy)] and [MoO_3_(Hbpdc)] (H_2_bpdc = 2,2′-bipyridine-5,5′-dicarboxylic acid), which display bands at 622/882/914 cm^−1^ and 576/901/922 cm^−1^, respectively [24,38]. An additional sharp band at 630 cm^−1^ for **2**^OD^ (not observed for **1**^OD^) is attributed to a 1,2,4-trz ring torsion vibration [35]. In a study of the infrared spectra of several transition metal 1,2,4-trz complexes, Haasnoot et al. determined that the presence of one ring torsion band in the 600–700 cm^–1^ range, typically around 630 cm^–1^, is indicative of 4-*H*,1,2-coordinating triazole rather than 1-*H*,2,4-coordinating triazole [35]. A similar band is observed for the layered hybrid [MoO_3_(1,2,4-trz)_0.5_] (**4**) (Figure 1h). Accordingly, in the region containing triazole ligand modes (980–1600 cm^−1^), the spectrum of **2**^OD^ closely resembles that of **4** (Figure 1g,h), suggesting that the ligand adopts a similar coordination mode. The structure of **4** [13,27] (and the isomorphous [MoO_3_(1,2,3-trz)_0.5_] (**3**) [28]) consists of layers of corner-sharing {MoO_5_N} octahedra composed of one terminal Mo=O group, four bridging Mo-O_b_ groups, and one N atom from a 1,2-bicoordinating triazole ligand that forms single [Mo^VI^(–(N–N)–Mo^VI^] bridges (Figure 2). The layered perovskite-like structure in **4** gives rise to two very broad *ν*(Mo‒O‒Mo) absorption bands near 600 and 840 cm^−1^, whereas **2**^OD^ (and **1**^OD^) only displays one band around 610 cm^−1^. Both **2**^OD^ and **4** exhibit a medium-intensity band near 1310 cm^−1^ that is assigned to a CH-bending vibration [35]. The shift of this band towards higher-energy (vs. 1272 cm^−1^ for solid 1,2,4-trz (Figure 1e) and 1275 cm^−1^ for **2** (Figure 1f)) is another marker for bidentate triazole [35]. From these observations we may tentatively propose chain-like structures for **1**^OD^ and **2**^OD^ consisting of corner-sharing {MoO_4_N_2_} octahedra composed of two terminal Mo=O groups, two bridging Mo-O_b_ groups, and two N atoms from two 1,2-bicoordinating triazole ligands that form single bridges between adjacent Mo^VI^ centers (Figure 2). This type of structural element has recently been identified in the trinuclear complex (1,2,4-Htrz)_2_[Mo_3_O_6_(O_2_)_4_(1,2,4-trz)_2_]⋅H_2_O (**6**) (Figure 2) [26]. The 1D chain-like structural motif is analogous to that found for [MoO_3_(2,2′-bipy)] [39] and [MoO_3_(Hbpdc)] [38], which contain {MoO_2_(*μ*_2_-O)_2_(bipy)} repeat units.

**Figure 3:**
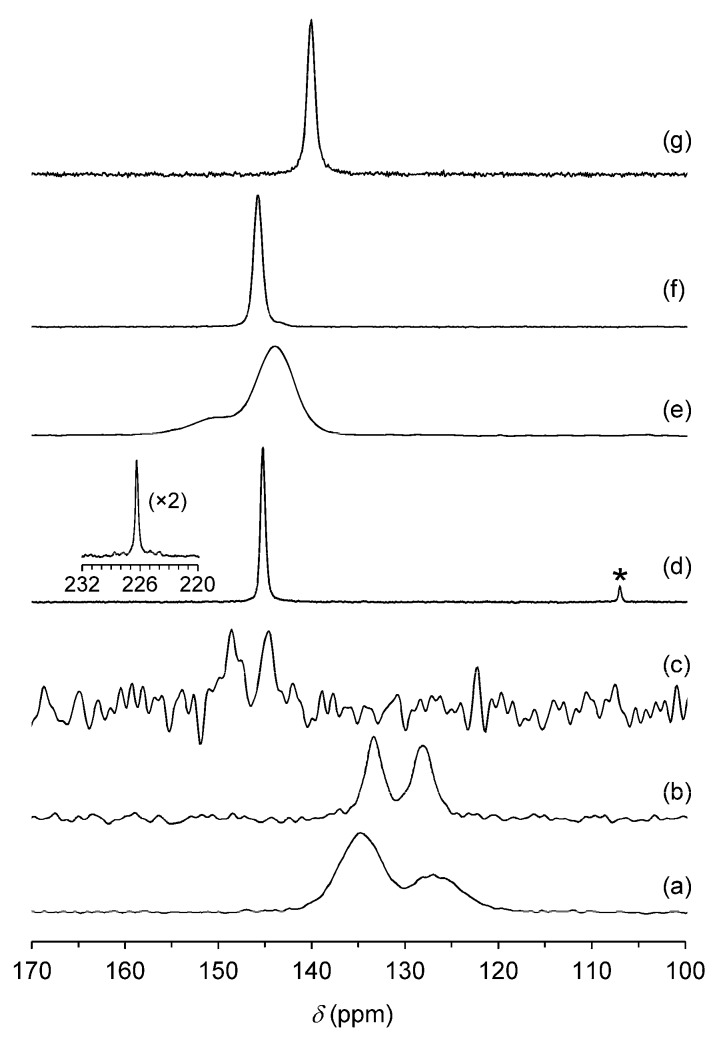
^13^C{^1^H} CP MAS NMR spectra of (**a**) oxidized derivative **1**^OD^, (**b**) hybrid **3**, (**c**) 1,2,4-trz, (**d**) complex **2**, (**e**) oxidized derivative **2**^OD^, (**f**) hybrid **4**, and (**g**) dinuclear complex **5**. The inset shows the single ^13^C resonance observed for the CO groups of **2** (**d**). The asterisk denotes a spinning sideband.

The ^13^C{^1^H} CP MAS NMR spectra of **1**^OD^ and **2**^OD^ provided further support for a bidentate bridging coordination of triazole molecules through the 1,2-nitrogen sites, analogous to that present in the hybrids **3** and **4** (Figure 3). As expected, the 1,2,3-trz layered hybrid **3** displays two peaks for the non-equivalent carbon atoms C4 (128.1 ppm) and C5 (133.3 ppm), while the 1,2,4-trz hybrid **4** displays a single peak at 145.5 ppm attributed to C3 and C5, which are chemically equivalent due to the N1,N2 bridging mode (Figure 3b,f). The spectra of **1**^OD^ and **2**^OD^ (Figure 3a,e) resemble those for **3** and **4**, respectively, since **1**^OD^ displays two peaks at 127.0 (C4) and 134.8 ppm (C5), while **2**^OD^ displays one main peak centered at 143.9 ppm (C3, C5). The observation of broader resonances for **1**^OD^ and **2**^OD^ when compared with **3** and **4** may be partly due to a higher degree of structural disorder for the former, since these solids were found by PXRD to be X-ray amorphous (not shown), while the layered hybrids are obtained as microcrystalline solids.

### 2.2. Dinuclear Complex (NH_4_)_1.8_(H_3_O)_0.2_[Mo_2_O_2_(μ_2_-O)(O_2_)_4_(1,2,4-trz)]·H_2_O *(**5**)*

The discovery that the layered hybrid [MoO_3_(1,2,4-trz)_0.5_] (**4**) could be used as a reaction-induced self-separating catalyst [13] emphasized the need to study the solution chemistry and to characterize oxo(peroxo)molybdenum(VI)-triazole complexes, which had not been previously reported. The first species to be isolated and structurally characterized was the yellow trinuclear complex (1,2,4-Htrz)_2_[Mo_3_O_6_(O_2_)_4_(1,2,4-trz)_2_] (**6**), which precipitated upon addition of 1,2,4-trz to the solution obtained by reaction of MoO_3_ with excess aq. H_2_O_2_ (in the absence of organic solvents) [26]. In the present work, further reactivity studies were performed in which **4** or **6** were treated with excess H_2_O_2_ in the presence of acetonitrile. Both reactions led to yellow solutions from which the dinuclear complex (NH_4_)_1.8_(H_3_O)_0.2_[Mo_2_O_2_(*μ*_2_-O)(O_2_)_4_(1,2,4-trz)]·H_2_O (**5**) crystallized. The FT-IR spectrum of **5** pointed to the unexpected formulation with the appearance of a strong band near 1400 cm^−1^ assigned to an NH bending vibration of NH_4_^+^ ions (Figure 1i). Two possible explanations for the presence of ammonium ions are the hydrolysis of CH_3_CN and the oxidative degradation of 1,2,4-trz. The IR spectrum of **5** displays additional diagnostic bands at 520, 579 and 625 cm^−1^ (*ν*[Mo(O_2_)_2_] and/or trz ring-torsion), 709 cm^−1^ [*ν*_asym_(Mo‒O‒Mo)], 864 cm^−1^ [*ν*(O‒O)], and 937 cm^−1^ [*ν*(Mo=O)]. Corresponding bands appear in the Raman spectrum at 542, 573, 636, 875 and 940 cm^−1^ (Figure S2 in the Supporting Information). The solid-state ^13^C{^1^H} CP MAS NMR spectrum of **5** displays one single sharp resonance at 140.0 ppm assigned to C3 and C5 carbon atoms (Figure 3g). The spectrum resembles that for the hybrid **4** (Figure 3f) except that in the former the resonance is shifted by 5.7 ppm towards higher field. These data point to the presence of a triazole N1,N2 bridging mode in **5**, analogous to that present in **4**, and this was subsequently confirmed by X-ray crystallographic studies.

The crystal structure of **5** reveals the presence of a centrosymmetric dinuclear anionic complex in which the mirror plane of the *P*nnm orthorhombic space group bisects the entire molecular unit. Within this mirror plane the crystal structure contains both the charge-balancing ammonium and hydronium cations as well as the water molecule of crystallization (not shown).

**Figure 4:**
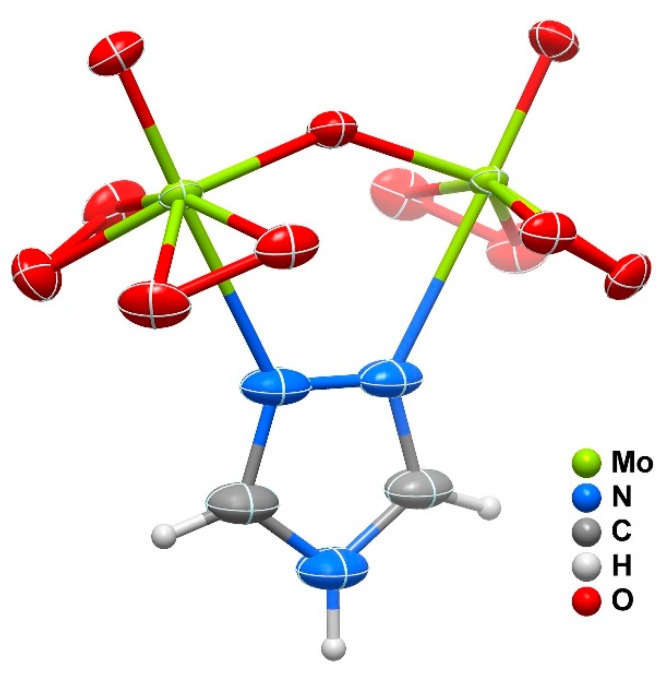
Schematic representation of the anionic dinuclear [Mo_2_O_2_(*μ*_2_-O)(O_2_)_4_(1,2,4-trz)]^2−^ complex present in the crystal structure of **5**. Non-hydrogen atoms are depicted as thermal ellipsoids drawn at the 50% probability level and hydrogen atoms as small spheres with arbitrary radii. Table S1 in the Supporting Information gives selected bond lengths and angles for the crystallographically independent Mo center.

The anionic [Mo_2_O_2_(*μ*_2_-O)(O_2_)_4_(1,2,4-trz)]^2−^ complex is built up from a single Mo^VI^ metal center which is coordinated to a *μ*_2_-bridging oxo group (also located on the mirror plane), a terminal oxo moiety, two peroxo ligands, and one nitrogen atom from a *N*,*N*-bridging triazole molecule as depicted in Figure 4. 

Taking the centers of gravity of the peroxo ligands as the coordinative positions, the overall coordination geometry resembles a slightly distorted bipyramid. Considering the equatorial plane of the polyhedron as being formed by the peroxo ligands and the *μ*_2_-bridging oxo group, the metal center appears raised towards the apical oxo moiety by ca. 0.35 Å. This latter group clearly exerts the well-known trans effect on the opposite coordinative bond to the N,N-bridging triazole molecule, with the refined Mo–N distance being 2.398(9) Å. This organic linker imposes a “kink” angle in the anionic dinuclear complex of 141.7(5)° and a Mo···Mo distance of 3.6731(5) Å.

### 2.3. Catalytic Studies

**Figure 5:**
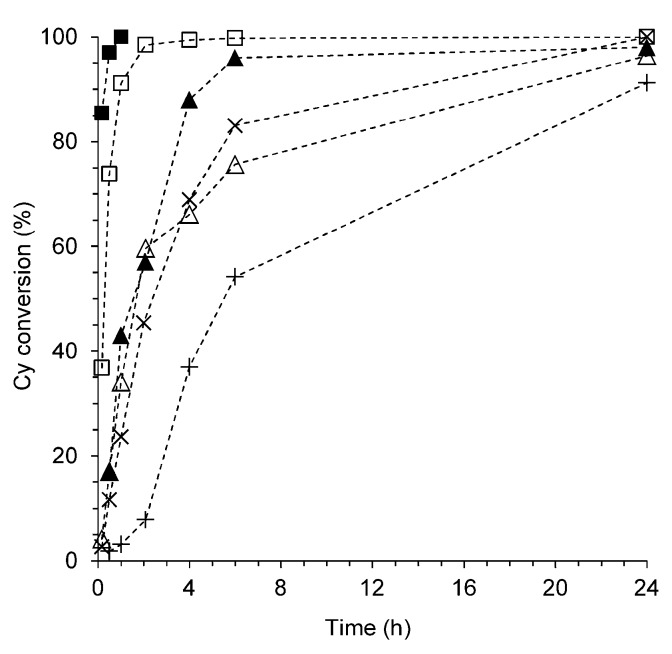
Kinetic profiles for the reaction of *cis*-cyclooctene with TBHP, in the presence of **1** (□), **2** (∆), **1**^OD^ (■), **2**^OD^ (▲), **3** (×) and **4** (+), at 70 °C. The CyO selectivity was always 100%. The dashed lines are visual guides.

The tricarbonyl complexes **1** and **2** were tested as catalyst precursors for the epoxidation of *cis*-cyclooctene (Cy) with TBHP at 70 °C, using α,α,α-trifluorotoluene (TFT) as a co-solvent (Table 1, Figure 5). Complex **1** bearing 1,2,3-trz led to a faster epoxidation reaction than complex **2** bearing 1,2,4-trz, with cyclooctene oxide (CyO) always being the only product formed (100% selectivity): **1** led to 99% conversion at 2 h, while **2** led to 60% conversion. The very favorable catalytic performance obtained using **1** is on a par with that reported previously for the complex [Mo(CO)_3_(HC(pz)_3_)] (HC(pz)_3_ = tris(1-pyrazolyl)methane), which led to 99% CyO yield after 2 h reaction at 55 °C (1,2-dichloroethane as co-solvent) [40]. The latter tricarbonyl complex was shown to undergo oxidative decarbonylation in situ, possibly leading to hexamolybdate species (Mo_6_O_19_^2−^) and/or an oxo-bridged dinuclear complex of the type [{MoO_2_(HC(pz)_3_)}_2_(*μ*-O)]^2+^. For complexes **1** and **2**, the ATR FT-IR spectra of undissolved solids recovered after batch catalytic runs of 24 h (denoted **1**^r^ and **2**^r^) very strongly resembled those of **1**^OD^ and **2**^OD^, suggesting that oxomolybdenum(VI)-triazole compounds were formed under the catalytic conditions and that these were structurally very similar to those obtained upon treatment of **1** and **2** with excess TBHP (Figure S3 in the Supporting Information).

The compounds **1**^OD^ and **2**^OD^ were tested as catalysts for Cy epoxidation with TBHP and were found to lead to faster reaction kinetics than the corresponding tricarbonyl precursors: **1**^OD^ led to 97% Cy conversion at 30 min, while **2**^OD^ led to 96% conversion at 6 h (Table 1, Figure 5). These results may be partly due to the involvement of an additional OD step when starting from the pre-catalysts, which is required for the formation of the active oxidizing species. This may retard the initial epoxidation reaction in relation to the direct use of the oxidized metal species (which avoids the in situ OD step). After batch catalytic runs of 24 h with **1**^OD^ and **2**^OD^, the resultant undissolved solids (**1**^ODr^ and **2**^ODr^) were recovered and characterized by ATR FT-IR spectroscopy (Figure S3 in the Supporting Information). The spectra were unchanged from those for the starting materials **1**^OD^ and **2**^OD^, showing that these underwent no further conversion to a structurally different solid phase. Nevertheless, for practical application, it may be important to check the long-term stability of these catalysts via catalytic and characterization studies, and develop catalyst separation and recovery strategies.

The catalytic performance of the oxidized compounds **1**^OD^ and **2**^OD^ was found to be greatly superior to that for the layered hybrids **3** and **4**: Compound **3** led to 83%/100% Cy conversion at 6 h/24 h, while **4** led to 54%/91% conversion (Table 1, Figure 5). In parallel with that observed for **1**^OD^ and **2**^OD^, ATR FT-IR spectra for the undissolved solids recovered after batch catalytic runs of 24 h (**3**^r^ and **4**^r^) did not differ significantly from those for the starting materials **3** and **4** (Figure S3 in the Supporting Information). The better catalytic activity of the 1,2,3-trz hybrid in relation to the 1,2,4-trz hybrid is in line with the results obtained with **1** and **2**, and **1**^OD^ and **2**^OD^. This apparent ligand influence on the epoxidation activity with TBHP may be partly due to solubility effects, with 1,2,3-trz leading to metal species with enhanced solubility.

The catalytic results for the most active compound **1**^OD^ (100% CyO yield at 1 h) compare favorably to literature data for hybrids of the general formula [MoO_3_(L_N_)] where L_N_ denotes an N-donor ligand. The [MoO_3_(L_N_)] hybrids that were tested under similar Cy reaction conditions to those used in the present work possessed imidazole or triazole-type L_N_ ligands, namely, [MoO_3_(pbim)] (pbim = 2-(2-pyridyl)-benzimidazole, 34%/72% CyO yield at 1 h/6 h) [41], [MoO_3_(*p*-trtzH)] (*p*-trtzH = 5-[4-(1,2,4-triazol-4-ylphenyl)]-1*H*-tetrazole, 4%/30%) [42] and [MoO_3_(*tr*NH_2_)]·0.26H_2_O (*tr*NH_2_ = 4-amino-1,2,4-triazole, 10%/45%) [42].

Compound **1**^OD^ was further investigated as a catalyst for the epoxidation of 1-octene, *trans*-2-octene, cyclohexene, cyclododecene and styrene, with TBHP at 70 °C (Table 2). For the linear olefins 1-octene and *trans*-2-octene, conversion at 6 h/24 h was 48%69% and 90%/99%, respectively, and epoxide selectivity was always 100%. *Trans*-2-Octene was more reactive than 1-octene, suggesting that internal C=C bonds are more prone to epoxidation than terminal C=C bonds. These electronic effects are somewhat consistent with mechanistic proposals reported in the literature for olefin epoxidation in the presence of molybdenum catalysts [43,44,45]. Specifically, the coordination reaction between the metal center and the oxidant molecule occurs with the concomitant protonation of an oxo ligand, forming an electrophilic intermediate species, which, in turn, is involved in an oxygen atom transfer reaction to the olefin. Thus, the higher nucleophilicity of the internal C=C bond (of *trans*-2-octene vs. 1-octene) is favorable to oxygen transfer. Compound **1**^OD^ was less active for the epoxidation of the bulkier, cyclic substrate cyclododecene than *cis*-cyclooctene (68 and 100% conversion at 1 h, respectively, and epoxide selectivity was always 100%), suggesting that steric effects may be important. These effects agree with the literature for different molybdenum catalysts [40,46]. With styrene as substrate, conversion at 6 h/24 h was 41%/52%; the products included styrene oxide and benzaldehyde formed with 2%/2% and 19%/25% yield, respectively. The poor epoxide selectivity may be partly due to the reactivity of styrene oxide.

The catalytic performance of all compounds for Cy epoxidation was subsequently studied using H_2_O_2_ as the oxidant instead of TBHP, with acetonitrile as the co-solvent instead of TFT (Table 1). With the notable exception of the layered hybrid **4**, the reaction kinetics was generally slower using H_2_O_2_ than TBHP, suggesting that the former oxidizing medium led to the formation of less active (and different) metal species. In contrast to that seen with TBHP as oxidant (comparing **1** vs. **2**, and **1**^OD^ vs. **2**^OD^), changing the ligand had no considerable influence on catalytic performance with H_2_O_2_, since **1** and **2** led to (75–77%)/(93–100%) conversion at 6 h/24 h, while **1**^OD^ and **2**^OD^ led to (53–64%)/(92–94%) conversion. For all four compounds the liquid phases of the H_2_O_2_-based reactions mixtures were yellow, while the TBHP-based ones were colorless. Attempts to isolate compounds from the yellow solutions were unsuccessful, possibly because the dissolved species were very strongly solvated. With the layered hybrids, **4** led to much better performance than **3** with H_2_O_2_ as oxidant, while the opposite was the case with TBHP. As described previously, the hybrid **4** displays unique behavior with H_2_O_2_ as a reaction-induced self-separating (RISS) catalyst [13]. Under the catalytic conditions, the RISS catalyst transforms into soluble metal species, giving a homogeneous mixture. Upon consumption of the oxidant, the soluble species convert reversibly into the original form of the catalyst, which precipitates and can be easily separated from the reaction mixture. Both **3** and **4** lead to yellow homogeneous solutions under the reaction conditions used for Cy epoxidation with H_2_O_2_. However, although **3** and **4** possess isomorphous solid-state structures, the hybrid **3** did not display RISS behavior, i.e., no precipitate formed upon completion of the reaction and consumption of the oxidant.

The yellow solutions formed by reaction of **3** and **4** with H_2_O_2_ under the Cy epoxidation conditions are assumed to contain discrete molecular oxoperoxomolybdenum(VI) complexes. In the case of 1,2,4-trz, these may only be stable in the presence of excess oxidant, i.e., the consumption of the oxidant promotes a reverse reaction in which the molecular species recombine to form the layered hybrid **4**. For 1,2,3-trz, this reverse reaction does not take place, which may be partly due to differences in the structure and solubility of the soluble active species, pointing to a significant ligand effect despite the subtle structural differences between the two isomeric triazoles. Attempts to isolate species from the solution obtained from **3** were not successful, suggesting that they may be strongly solvated. As mentioned above, we were previously successful in isolating the trinuclear complex **6** from a MoO_3_/1,2,4-trz/H_2_O_2_ mixture [26]. The catalytic performance of **6** for Cy epoxidation with H_2_O_2_ at 70 °C in the presence of CH_3_CN (92%/99% CyO yield at 6 h/24 h) was comparable with that for **4**, and accordingly it was found that the undissolved solid present at the end of the catalytic reaction was microcrystalline **4**, i.e., **6** converted in situ to **4** [26]. These observations prompted our studies in the present work on the treatment of **4** and **6** with excess aq. H_2_O_2_ in the presence of CH_3_CN, which led to the isolation of the dinuclear complex **5** from yellow solutions. The catalytic performance of complex **5** for Cy epoxidation with aq. H_2_O_2_ at 70 °C (39% CyO yield at 24 h) was inferior to that for **4** and **6** (90–92% yield). Nevertheless, these studies suggest that dinuclear or trinuclear oxoperoxomolybdenum(VI) complexes bearing bridging triazole ligands are likely to be the homogeneous catalysts responsible for activating the oxidant for olefin epoxidation when using the hybrids **3** and **4** as pre-catalysts.

## 3. Materials and Methods

### 3.1. Materials

Reagents (Mo(CO)_6_, 1*H*-1,2,3-triazole (97%, Manchester Organics, Runcorn, UK), 1*H*-1,2,4-triazole (98%), 5–6 M *tert*-butyl hydroperoxide in decane, anhydrous dichloromethane, toluene (99.9%), acetone (99.5%), diethyl ether (99.8%) and pentane (≥95%, Carlo Erba, Milan, Italy) were purchased from Sigma-Aldrich (St. Louis, MO, USA) unless otherwise indicated. For the catalytic experiments, *cis*-cyclooctene (95%), acetonitrile (≥99.9%), anhydrous α,α,α-trifluorotoluene (99%), pentane (≥99.5%) and 30% aq. H_2_O_2_ were acquired from Sigma-Aldrich and used as received. The hybrid metal oxide-triazole material [MoO_3_(1,2,4-trz)_0.5_] (**4**) was prepared by the hydrothermal reaction of MoO_3_, 1,2,4-trz and water at 180 °C for 1 h [13]. The phase purity of the microcrystalline product was verified by a comparison of its powder X-ray diffraction (PXRD) pattern with a simulated pattern calculated using the crystal structure data published for **4** (Figure S4 in the Supporting Information) [13]. The trinuclear complex (1,2,4-Htrz)_2_[Mo_3_O_6_(O_2_)_4_(1,2,4-trz)_2_]⋅H_2_O (**6**) was prepared as described previously by the addition of 1,2,4-trz to the solution obtained upon reaction of MoO_3_ with a large excess of H_2_O_2_ [26].

### 3.2. Methods

Microanalyses for C, H and N were carried out at the Department of Chemistry, University of Aveiro, with a TruSpec Micro CHNS 630-200-200 elemental analyser (Leco, Saint Joseph, MI, USA). TGA was performed using a TGA-50 system (Shimadzu, Kyoto, Japan) at a heating rate of 5 °C min^−1^ under air. PXRD data were collected at ambient temperature using an Empyrean instrument (Malvern Panalytical, Malvern, UK) equipped with a PIXcel 1D detector set at 240 mm from the sample. Cu-Kα_1,2_ X-radiation (*λ*_1_ = 1.540598 Å; *λ*_2_ = 1.544426 Å) filtered with a nickel foil was used, with the X-ray tube operating at 45 kV and 40 mA. Samples were supported in a spinning flat plate sample holder and intensity data were collected by the step-counting method (step 0.02°) in continuous mode. FT-IR spectra were recorded on a 7000 FT-IR spectrometer (Mattson Instruments Inc., Madison, WI, USA) using KBr pellets. Attenuated total reflectance (ATR) FT-IR spectra were measured on the same instrument using a Golden Gate Mk II ATR accessory (Specac, Orpington, UK) having a diamond top plate and KRS-5 focusing lenses. FT-Raman spectra were recorded on a RFS-100 FT-Spectrometer (Bruker, Billerica, MA, USA) equipped with a Nd:YAG laser with an excitation wavelength of 1064 nm. ^1^H-NMR spectra were recorded on an Avance 300 spectrometer (Bruker, Billerica, MA, USA). Samples for NMR were prepared in a glove box, maintained under Ar, and measured immediately. Chemical shifts are quoted in ppm from TMS. Solid-state ^13^C{^1^H} CP MAS NMR spectra were recorded using a Bruker Avance 400 (narrow bore) spectrometer with an ultrashielded static magnetic field of 100.6 MHz. The spectra were recorded with 2.75 μs ^1^H 90° pulses, 2.0 ms contact time, spinning rate of 14 kHz, and 5 s recycle delays. The spectrum for **2** was recorded with 3.2 μs ^1^H 90° pulses, 6.5 ms contact time, spinning rate of 12 kHz, and 5 s recycle delays. Chemical shifts are quoted in ppm from TMS.

### 3.3. Synthetic Procedures

#### 3.3.1. [Mo(CO)_3_(1,2,3-trz)_3_] (**1**)

Mo(CO)_6_ (0.50 g, 1.90 mmol) and 1*H*-1,2,3-trz (0.34 mL, 5.70 mmol) were added to toluene (20 mL) in a Schlenk tube and the mixture was refluxed under N_2_ for 90 min, resulting in a dark green precipitate and a yellow solution. The solid was filtered, washed with a 1:1 mixture of pentane and diethyl ether (2 × 20 mL), and vacuum-dried. Yield: 0.60 g, 82%. Anal. Calcd for C_9_H_9_MoN_9_O_3_ (387.17): C, 27.92; H, 2.34; N, 32.56. Found: C, 27.35; H, 2.15; N, 32.64%. Selected FT-IR bands (KBr, cm^−1^): 476 (w), 523 (m), 700 (w), 735 (w), 787 (m), 808 (m), 833 (w), 958 (w), 1070 (w), 1088 (m), 1155 (w), 1198 (w), 1448 (w), 1527 (m), 1774 (vs,bd) [*ν*(CO)], 1907 (s) [*ν*(CO)], 3140 (m), 3165 (m), 3311 (m). ^1^H-NMR ((CD_3_)_2_CO, r.t., Figure S5 in the Supporting Information): *δ* = 7.79 (br, s), 8.24 (s), 8.37 (d), 14.45 (vbr, sh), 15.06 (vbr, s). A tentative assignment of the ^1^H-NMR spectrum is based on the coexistence of Mo-coordinated 1*H* (*T*-1*H*) and 2*H* (*T*-2*H*) annular tautomers of 1,2,3-trz, in correspondence with that known to exist for free 1,2,3-trz in the solid, liquid and gas phases [47,48]: *δ* (*T*-2*H*) 7.79 (2H, H4 and H5), 14.45 (1H, NH); δ (*T*-1*H*) 8.24 (1H, H4 or H5), 8.37 (1H, H4 or H5), 15.06 (1H, NH); approximate *T*-2*H*:*T*-1*H* ratio = 1:1.8. TGA (Figure S1 in the Supporting Information) showed a residual mass of 37.4% at 450 °C (Calcd for MoO_3_: 37.2%).

#### 3.3.2. [Mo(CO)_3_(1,2,4-trz)_3_] (**2**)

Mo(CO)_6_ (0.25 g, 0.95 mmol) and 1*H*-1,2,4-trz (0.20 g, 2.85 mmol) were added to toluene (10 mL) in a Schlenk tube and the mixture was refluxed under N_2_ for 2 h. The resultant cream-colored precipitate was filtered, washed with acetone (2 × 20 mL) and diethyl ether (2 × 20 mL), and vacuum-dried. Yield: 0.34 g, 95%. Anal. Calcd for C_9_H_9_MoN_9_O_3_ (387.17): C, 27.92; H, 2.34; N, 32.56. Found: C, 27.99; H, 2.45; N, 32.88%. Selected FT-IR bands (KBr, cm^−1^): 482 (w), 517 (w), 527 (w), 605 (m), 699 (w), 859 (w), 879 (w), 947 (w), 1031 (m), 1125 (m), 1296 (m), 1410 (m), 1425 (m), 1512 (m), 1760 (vs,bd) [*ν*(CO)], 1905 (s) [*ν*(CO)], 3138 (w), 3159 (m), 3170 (m), 3382 (m). ^13^C{^1^H} CP MAS NMR: *δ* = 145.2 (C3, C5), 226.3 (CO). TGA (Figure S1 in the Supporting Information) showed a residual mass of 36.3% at 450 °C (Calcd for MoO_3_: 37.2%).

#### 3.3.3. Oxidative Decarbonylation Reactions

Oxidative decarbonylation of **1** or **2** was performed in a Schlenk tube under nitrogen by addition of 5–6 M TBHP in decane to a suspension of the tricarbonyl complex in CH_2_Cl_2_. Reaction conditions for **1**: TBHP (2.0 mL, ca. 10.9 mmol), **1** (0.35 g, 1.11 mmol), CH_2_Cl_2_ (45 mL), 4 h, r.t. The resultant light violet solid (**1**^OD^) was recovered by filtration, washed with diethyl ether (2 × 45 mL), and vacuum-dried. Yield: 0.19 g (74%). Reaction conditions for **2**: TBHP (2.1 mL, ca. 11.4 mmol), **2** (0.12 g, 0.38 mmol), CH_2_Cl_2_ (20 mL), 70 °C, 48 h. The resultant light brown solid (**2**^OD^) was filtered, washed with CH_2_Cl_2_ (2 × 20 mL) and acetone (2 × 20 mL), and vacuum-dried. Yield: 0.07 g.

Data for **1**^OD^: Anal. Calcd for C_2_H_3_MoN_3_O_3_·H_2_O (231.02): C, 10.40; H, 2.18; N, 18.19. Found: C, 9.86; H, 1.82; N, 17.53%. Residual masses recorded by TGA (Figure S1 in the Supporting Information) were 6.3% at 110 °C (Calcd for H_2_O: 7.8%) and 62.0% at 400 °C (Calcd for MoO_3_: 62.3%). FT-IR (KBr, cm^−1^): 615 (vbd) [*ν*_as_(Mo‒O‒Mo)], 796 (m), 902 (vs) [*ν*(Mo=O)], 941 (s) [*ν*(Mo=O)], 974 (m), 1092 (m), 1132 (m), 1205 (m), 1260 (w), 1446 (m), 2870 (w), 3140 (m). ^13^C{^1^H} CP MAS NMR: *δ* = 127.0 (C4), 134.8 (C5).

Data for **2**^OD^: C, 10.88; H, 2.09; N, 17.03%. FT-IR (KBr, cm^−1^): 610 (vbd) [*ν*_as_(Mo‒O‒Mo)], 630 (m), 899 (vs) [*ν*(Mo=O)], 936 (s) [*ν*(Mo=O)], 1059 (m), 1153 (m), 1212 (w), 1310 (m), 1385 (w), 1413 (m), 1510 (m), 1528 (sh), 1628 (w), 2814 (w), 2895 (w), 2977 (w), 3128 (m), 3440 (vbd). ^13^C{^1^H} CP MAS NMR: *δ* = 143.9 (C3, C5).

#### 3.3.4. [MoO_3_(1,2,3-trz)_0.5_] (**3**)

A mixture of MoO_3_ (0.43 g, 3 mmol), 1,2,3-trz (0.18 mL, 3 mmol) and water (20 mL) was heated in a static Teflon-lined stainless steel digestion bomb at 180 °C for 48 h. After cooling down to ambient temperature, the resultant black precipitate was filtered and washed with water (2 × 10 mL), acetone (2 × 10 mL) and diethyl ether (2 × 10 mL), and vacuum-dried. Yield: 0.29 g, 55%. TGA (Figure S1 in the Supporting Information) showed a residual mass of 83.0% at 400 °C (Calcd for MoO_3_: 80.6%). FT-IR (KBr, cm^−1^): 580 (vbd), 675 (vbd), 769 (m), 920 (sh), 938 (sh), 953 (sh), 962 (s), 1112 (s), 1150 (s), 1260 (w). A satisfactory match was obtained between the PXRD pattern of the microcrystalline product and a simulated pattern calculated using the crystallographic data published for the structure of [MoO_3_(1,2,3-trz)_0.5_] (Figure S4 in the Supporting information) [28]. ^13^C{^1^H} CP MAS NMR: *δ* = 128.1 (C4), 133.3 (C5).

#### 3.3.5. (NH_4_)_1.8_(H_3_O)_0.2_[Mo_2_O_2_(μ_2_-O)(O_2_)_4_(1,2,4-trz)]·H_2_O (**5**)

Trinuclear **6** (0.20 g, 0.24 mmol) in CH_3_CN (45 mL) was treated with 30% H_2_O_2_ (12.75 mL, 125 mmol) and the resultant yellow solution was stirred at 70 °C for 24 h. After concentrating the solution under reduced pressure, hexane (10 mL) and diethyl ether (20 mL) were added, followed by dropwise addition of acetone to precipitate **5** as a dark yellow microcrystalline solid, which was recovered by filtration and vacuum-dried (0.13 g, 71% based on Mo). Single-crystals of **5** suitable for XRD were obtained by addition of a small quantity (ca. 1 mL) of acetone to the CH_3_CN/H_2_O mother liquor (2 mL), followed by slow evaporation at ambient temperature. Anal. Calcd for C_2_H_12.8_Mo_2_N_4.8_O_12.2_ (491.23): C, 4.89; H, 2.63; N, 13.69. Found: C, 4.67; H, 2.99; N, 13.76. TGA (Figure S1 in the Supporting Information) showed a residual mass of 58.1% at 400 °C (Calcd for MoO_3_: 58.6%). A satisfactory match was obtained between the PXRD pattern of the microcrystalline product and a simulated pattern calculated using the crystal structure data reported in this paper (Figure S4 in the Supporting information). FT-IR (KBr, cm^−1^): 520 (m) (*ν*_sym_[Mo(O_2_)_2_]), 579 (s) (*ν*_asym_[Mo(O_2_)_2_]), 625 (s) (*ν*[Mo(O_2_)_2_] and/or trz ring-torsion), 691 (m), 709 (vs) [*ν*_asym_(Mo‒O‒Mo)], 864 (vs) [*ν*(O‒O)], 937 (vs) [*ν*(Mo=O)], 1059 (m), 1125 (w), 1305 (w), 1403 (vs) [*δ*(NH_4_^+^)], 1496 (w), 1526 (w), 1604 (w), 1672 (w), 3125 (m) [*ν*(C‒H)], 3220 (bd), 3621 (m). Raman (cm^−1^): 314 (m), 419 (w), 542 (s) (*ν*_sym_[Mo(O_2_)_2_]), 573 (m) (*ν*_asym_[Mo(O_2_)_2_]), 636 (w), 875 (m) [*ν*(O‒O)], 940 (vs) [*ν*(Mo=O)], 1056 (w), 1125 (w), 1305 (m), 1401 (vw). ^13^C{^1^H} CP MAS NMR: *δ* = 140.0 (C3, C5).

Alternative synthesis of **5**: 30% H_2_O_2_ (4.4 mL, 43 mmol) was added to [MoO_3_(1,2,4-trz)_0.5_] (**4**) (0.05 g, 0.28 mmol) and CH_3_CN (15 mL) in a Schlenk tube. The suspension was stirred for 24 h at 70 °C. After cooling to room temperature, the resultant yellow solution was concentrated under reduced pressure and stored under nitrogen. After 2 weeks, the mother liquor was filtered off and allowed to evaporate slowly at ambient temperature under air, leading to yellow crystals of **5** (confirmed by PXRD). Yield: 0.03 g, 37%.

### 3.4. Single-Crystal X-ray Diffraction Studies

Single crystals of (NH_4_)_1.8_(H_3_O)_0.2_[Mo_2_O_2_(μ_2_-O)(O_2_)_4_(1,2,4-trz)]·H_2_O (**5**) were manually harvested from the crystallization vial and immersed in highly viscous FOMBLIN Y perfluoropolyether vacuum oil (LVAC 140/13, Sigma-Aldrich) to avoid degradation caused by the evaporation of the solvent [49]. Crystals were mounted on Hampton Research CryoLoops with the help of a Stemi 2000 stereo microscope (Carl Zeiss, Oberkochen, Germany). X-ray diffraction data for **5** were collected on a Bruker D8 QUEST at 150(2) K equipped with a Mo-Kα sealed tube (*λ* = 0.71073 Å), a multilayer TRIUMPH X-ray mirror, a PHOTON 100 CMOS detector, and a Cryostream 700+ Series low temperature device (Oxford Instruments, Abingdon, UK).

Diffraction images were processed using the software package SAINT+ (Bruker, Billerica, MA, USA) [50], and data were corrected for absorption by the multiscan semi-empirical method implemented in SADABS [51]. The structure was solved using the algorithm implemented in SHELXT-2014/5 [52], which allowed the immediate location of almost all of the heaviest atoms composing the molecular unit. The remaining missing and misplaced non-hydrogen atoms were located from difference Fourier maps calculated from successive full-matrix least-squares refinement cycles on *F*^2^ using the latest SHELXL from the 2018/3 release [53,54,55]. All structural refinements were performed using the graphical interface ShelXle [56].

Hydrogen atoms belonging to the organic linker were placed at their idealized positions using the *HFIX 43* instructions in SHELXL. These hydrogen atoms were included in subsequent refinement cycles with isotropic thermal displacement parameters (*U*_iso_) fixed at 1.2× *U*_eq_ of the parent atoms.

Hydrogen atoms associated with the NH_4_^+^, H_3_O^+^ and H_2_O moieties were directly located from difference Fourier maps. These hydrogen atoms were included in the final structural model with the N–H and O–H distances restrained to 0.95(1) Å, and with the isotropic thermal displacement parameters (*U*_iso_) fixed at 1.5× *U*_eq_ of the parent atoms. The intra-unit H···H distances were further restrained to 1.55(1) Å in order to ensure chemically reasonable geometries for these moieties.

The last difference Fourier map synthesis showed the highest peak (1.592 eÅ^−3^) and the deepest hole (−2.048 eÅ^−3^) located at 0.06 and 0.08 Å from N1 and N2, respectively. Structural drawings have been created using the software package Diamond (Crystal Impact, Bonn, Germany) [57].

Crystal data: C_2_H_12.8_Mo_2_N_4.8_O_12.2_, *M* = 491.23, orthorhombic, space group *P*nnm, *Z* = 4, *a* = 11.3410(15) Å, *b* = 13.0981(17) Å, *c* = 8.8302(12) Å, *V* = 1311.7(3) Å^3^, *μ*(Mo-Kα) = 1.989 mm^−1^, *D*_c_ = 2.488 mg cm^−3^, yellow block, crystal size of 0.22 × 0.10 × 0.10 mm^3^. Of a total of 25,388 reflections collected, 1261 were independent (*R*_int_ = 0.0713). Final *R*1 = 0.0614 [*I* > 2*σ(I)*] and *wR*2 = 0.1338 (all data). Data completeness to theta = 25.24°, 98.0%.

Crystallographic data (including structure factors) for the crystal structure of **5** have been deposited with the Cambridge Crystallographic Data Centre (CCDC) as supplementary publication number CCDC-1877431. Copies of the data can be obtained online at:  https://www.ccdc.cam.ac.uk/structures/.

### 3.5. Catalytic Tests

The epoxidation reaction of the model olefin substrate *cis*-cyclooctene (Cy) was carried at 70 °C using a temperature-controlled oil bath. Typically, an initial molybdenum catalyst/substrate molar ratio of 0.01, an oxidant/substrate molar ratio of 0.65 (1.8 mmol substrate), and 2 mL of cosolvent were used. Reactions with TBHP were performed with α,α,α-trifluorotoluene (TFT) as the cosolvent in 10 mL borosilicate reactors equipped with a valve for sampling. The reaction mixture (catalyst, olefin and solvent) was preheated to 70 °C under magnetic stirring (PTFE-coated stirring bar) for 10 min, and then the oxidant (also preheated under similar conditions in a separate vessel) was added. Samples were taken at different instants from the same reactor. After the catalytic tests, solid phases were isolated by centrifugation (3500 rpm), thoroughly washed with diethyl ether, pentane or acetone, and vacuum-dried at 60 °C for 1 h. With H_2_O_2_ as the oxidant, CH_3_CN was used as the cosolvent and the catalytic tests were carried with magnetic stirring in tubular borosilicate reactors with pear-shaped bottoms (ca. 12 mL of capacity), equipped with a valve for sampling. Separate catalytic experiments were carried out for each reaction time, and the reactors were cooled to ambient temperature prior to sampling.

The evolution of the catalytic reactions was monitored by gas chromatography. The samples were analyzed using a Varian 3900 GC (Agilent Technologies, Santa Clara, CA, USA) equipped with a DB-5 capillary column (30 m × 0.25 mm × 0.25 µm) and a FID detector, with H_2_ as the carrier gas. The identification of the reaction products was performed by GC-MS (GC-qMS 6890 N Network GC system, 5973 Network mass selective detector, Agilent Technologies), equipped with a DB-1 capillary column (30 m × 0.25 mm × 0.10 µm), using He as the carrier gas, and quantifications were based on calibrations.

## 4. Conclusions

The main advances achieved in the present work can be summarized as follows: (i) synthesis of molybdenum tricarbonyl complexes with the two isomeric unsubstituted triazoles; (ii) use of the tricarbonyl complexes as precursors to molybdenum oxide-triazole hybrids; (iii) demonstration of ligand effects in Mo-catalyzed olefin epoxidation using either the tricarbonyl precursors directly or the oxidized derivatives; (iv) synthesis and X-ray structure determination of a new oxoperoxomolybdenum(VI) complex bearing 1,2,4-triazole ligands, which provides a valuable insight into the type of solution species that may be involved in the H_2_O_2_-based processes with Mo-triazole catalysts. Based on the results obtained, molybdenum compounds with 1,2,3-triazole are promising and warrant further investigation for a broader range of oxidation reactions such as sulfoxidation and the oxidation of amines. We hope to pursue these studies in future work.

## Figures and Tables

**Table 1:** Catalytic results for the epoxidation of *cis*-cyclooctene ^1^.

Compound	Oxidant	Conversion or Epoxide Yield at 1 h/6 h/24 h (%) ^2^
**1**	TBHP	91/100/-
**2**	TBHP	34/76/96
**1** ^OD^	TBHP	100/-/-
**2** ^OD^	TBHP	43/96/98
**3**	TBHP	24/83/100
**4**	TBHP	3/54/91
**1**	H_2_O_2_	-/77/93
**2**	H_2_O_2_	-/75/100
**1** ^OD^	H_2_O_2_	-/53/92
**2** ^OD^	H_2_O_2_	-/64/94
**3**	H_2_O_2_	-/62/73
**4**	H_2_O_2_	-/90/98 ^3^

^1^ Reaction conditions: Initial molar ratio Mo:Cy:oxidant = 1:100:153, [Cy]_0_ = 1.0 M, 2 mL co-solvent (TFT for TBHP and CH_3_CN for H_2_O_2_), 70 °C. ^2^ Cyclooctene oxide (CyO) yield (selectivity was 100%). Blank reactions (no catalyst): Less than 7% CyO yield at 24 h with TBHP; negligible Cy reaction with H_2_O_2_. ^3^ Data from Ref. [13].

**Table 2:** Catalytic epoxidation of olefins with TBHP, in the presence of **1**^OD 1^.

Substrate	Main Product	Conversion at 1 h/6 h/24 h (%)	Epoxide Yield at 1 h/6 h/24 h (%)
*cis*-cyclooctene	epoxide	100/-/-	100/-/-
1-octene	epoxide	15/48/69	15/48/69
*trans*-2-octene	epoxide	70/90/99	70/90/99
cyclododecene	epoxide	68/98/100	68/98/100
styrene	benzaldehyde	23/41/52	14/6/3

^1^ Reaction conditions: Initial molar ratio Mo:substrate:oxidant = 1:100:153, [substrate]_0_ = 1.0 M, 2 mL TFT, 70 °C.

## Supplementary Materials

The following are available online. Figure S1: TGA curves for **1**, **2**, **1**^OD^, **3** and **5**, Figure S2: Raman spectrum of **5**, Figure S3: ATR FT-IR spectra of **1**^OD^, **1**^ODr^, **1**^r^, **2**^OD^, **2**^ODr^, **2**^r^, **3**, **3**^r^, **4** and **4**^r^, Figure S4: Computed and experimental PXRD patterns of **3**, **4** and **5**, Figure S5: ^1^H NMR spectrum of **1**, Table S1: Selected bond distances and angles for the molecular structure of **5**.

## References

[1] Sanz R., Pedrosa M.R. (2009). Applications of Dioxomolybdenum(VI) Complexes to Organic Synthesis. Curr. Org. Synth..

[2] Amini M., Haghdoost M.M., Bagherzadeh M. (2013). Oxido-peroxido molybdenum(VI) complexes in catalytic and stoichiometric oxidations. Coord. Chem. Rev..

[3] Montilla F., Galindo A., Reedijk J. (2017). Oxidodiperoxidomolybdenum Complexes: Properties and Their Use as Catalysts in Green Oxidations. Elsevier Reference Module in Chemistry, Molecular Sciences and Chemical Engineering.

[4] Kühn F.E., Santos A.M., Abrantes M. (2006). Mononuclear Organomolybdenum(VI) Dioxo Complexes: Synthesis, Reactivity, and Catalytic Applications. Chem. Rev..

[5] Wang S.-S., Yang G.-Y. (2015). Recent Advances in Polyoxometalate-Catalyzed Reactions. Chem. Rev..

[6] Abrantes M., Santos A.M., Mink J., Kühn F.E., Romão C.C. (2003). A Simple Entry to (*η*^5^-C_5_R_5_)chlorodioxomolybdenum(VI) Complexes (R = H, CH_3_, CH_2_Ph) and Their Use as Olefin Epoxidation Catalysts. Organometallics.

[7] Barlan A.U., Basak A., Yamamoto H. (2006). Enantioselective Oxidation of Olefins Catalyzed by a Chiral Bishydroxamic Acid Complex of Molybdenum. Angew. Chem. Int. Ed..

[8] Zong L., Wang C., Moeljadi A.M.P., Ye X., Ganguly R., Li Y., Hirao H., Tan C.-H. (2006). Bisguanidinium dinuclear oxodiperoxomolybdosulfate ion pair-catalyzed enantioselective sulfoxidation. Nat. Commun..

[9] Romano F., Linden A., Mba M., Zonta C., Licini G. (2010). Molybdenum(VI) Amino Triphenolate Complexes as Catalysts for Sulfoxidation, Epoxidation and Haloperoxidation. Adv. Synth. Catal..

[10] Meninno S., Parrella A., Brancatelli G., Geremia S., Gaeta C., Talotta C., Neri P., Lattanzi A. (2015). Polyoxomolybdate-Calix[4]arene Hybrid: A Catalyst for Sulfoxidation Reactions with Hydrogen Peroxide. Org. Lett..

[11] Shylesh S., Schweizer J., Demeshko S., Schünemann V., Ernst S., Thiel W.R. (2009). Nanoparticle Supported, Magnetically Recoverable Oxodiperoxo Molybdenum Complexes: Efficient Catalysts for Selective Epoxidation Reactions. Adv. Synth. Catal..

[12] Zhou M.-D., Liu M.-J., Huang L.-L., Zhang J., Wang J.-Y., Li X.-B., Kühn F.E., Zang S.-L. (2015). Olefin epoxidation with hydrogen peroxide using octamolybdate-based self-separating catalysts. Green Chem..

[13] Amarante T.R., Neves P., Valente A.A., Paz F.A.A., Pillinger M., Gonçalves I.S. (2016). Metal oxide-triazole hybrids as heterogeneous or reaction-induced self-separating catalysts. J. Catal..

[14] Jimtaisong A., Luck R.L. (2006). Synthesis and Catalytic Epoxidation Activity with TBHP and H_2_O_2_ of Dioxo-, Oxoperoxo-, and Oxodiperoxo Molybdenum(VI) and Tungsten(VI) Compounds Containing Monodentate or Bidentate Phosphine Oxide Ligands: Crystal Structures of WCl_2_(O)_2_(OPMePh_2_)_2_, WCl_2_(O)(O_2_)(OPMePh_2_)_2_, MoCl_2_(O)_2_dppmO_2_.·C_4_H_10_O, WCl_2_(O)_2_dppmO_2_, Mo(O)(O_2_)_2_dppmO_2_, and W(O)(O_2_)_2_dppmO_2_. Inorg. Chem..

[15] Al-Ajlouni A.M., Veljanovski D., Capapé A., Zhao J., Herdtweck E., Calhorda M.J., Kühn F.E. (2009). Kinetic Studies on the Oxidation of η^5^-Cyclopentadienyl Methyl Tricarbonyl Molybdenum(II) and the Use of Its Oxidation Products as Olefin Epoxidation Catalysts. Organometallics.

[16] Veiros L.F., Gamelas C.A., Calhorda M.J., Romão C.C. (2011). Chemoselective Sulfide and Sulfoxide Oxidations by CpMo(CO)_3_Cl/HOOR: A DFT Mechanistic Study. Organometallics.

[17] Drees M., Hauser S.A., Cokoja M., Kühn F.E. (2013). DFT studies on the reaction pathway of the catalytic olefin epoxidation with CpMoCF_3_ dioxo and oxo–peroxo complexes. J. Organomet. Chem..

[18] Hauser S.A., Reich R.M., Mink J., Pöthig A., Cokoja M., Kühn F.E. (2015). Influence of structural and electronic properties of organomolybdenum(II) complexes of the type [CpMo(CO)_3_R] and [CpMo(O_2_)(O)R] (R = Cl, CH_3_, CF_3_) on the catalytic olefin epoxidation. Catal. Sci. Technol..

[19] Reich R.M., Kaposi M., Pöthig A., Kühn F.E. (2016). Kinetic studies of fluorinated aryl molybdenum(II) tricarbonyl precursors in epoxidation catalysis. Catal. Sci. Technol..

[20] Brégeault J.-M. (2003). Transition-metal complexes for liquid-phase catalytic oxidation: Some aspects of industrial reactions and of emerging technologies. Dalton Trans..

[21] Martins A.M., Romão C.C., Abrantes M., Azevedo M.C., Cui J., Dias A.R., Duarte M.T., Lemos M.A., Lourenço T., Poli R. (2005). Mononuclear and Binuclear Cyclopentadienyl Oxo Molybdenum and Tungsten Complexes: Syntheses and Applications in Olefin Epoxidation Catalysis. Organometallics.

[22] Li S., Kee C.W., Huang K.-W., Hor T.S.A., Zhao J. (2010). Cyclopentadienyl Molybdenum(II/VI) N-Heterocyclic Carbene Complexes: Synthesis, Structure, and Reactivity under Oxidative Conditions. Organometallics.

[23] Neves P., Amarante T.R., Gomes A.C., Coelho A.C., Gago S., Pillinger M., Gonçalves I.S., Silva C.M., Valente A.A. (2011). Heterogeneous oxidation catalysts formed in situ from molybdenum tetracarbonyl complexes and *tert*-butyl hydroperoxide. Appl. Catal. A Gen..

[24] Amarante T.R., Neves P., Coelho A.C., Gago S., Valente A.A., Paz F.A.A., Pillinger M., Gonçalves I.S. (2010). Investigation of Molybdenum Tetracarbonyl Complexes as Precursors to Mo^VI^ Catalysts for the Epoxidation of Olefins. Organometallics.

[25] Neves P., Nogueira L.S., Valente A.A., Pillinger M., Gonçalves I.S., Sampaio-Dias I.E., Sousa C.A.D., Rizzo-Aguiar F., Rodríguez-Borges J.E. (2018). Performance of chiral tetracarbonylmolybdenum pyrindanyl amine complexes in catalytic olefin epoxidation. J. Organomet. Chem..

[26] Antunes M.M., Amarante T.R., Valente A.A., Paz F.A.A., Gonçalves I.S., Pillinger M. (2018). A linear trinuclear oxidodiperoxido-molybdenum(VI) complex with single triazole bridges: Catalytic activity in epoxidation, alcoholysis, and acetalization reactions. ChemCatChem.

[27] Hagrman P.J., LaDuca R.L., Koo H.J., Rarig R., Haushalter R.C., Whangbo M.H., Zubieta J. (2000). Ligand Influences on the Structures of Molybdenum Oxide Networks. Inorg. Chem..

[28] Chuang J., Oullette W., Zubieta J. (2008). Solid state coordination chemistry of molybdenum oxides with 1,2,3-triazole (Htrz): The crystal structures of [Cu(I)Cu(II)_2_(trz)_2_Mo_4_O_13_(OH)], [MoO_3_(Htrz)_0.5_] and [Cu(I)trz]. Inorg. Chim. Acta.

[29] Huang D., Zhao P., Astruc D. (2014). Catalysis by 1,2,3-triazole- and related transition-metal complexes. Coord. Chem. Rev..

[30] Zurro M., Mancheño O.G. (2017). 1,2,3,–Triazole-Based Catalysts: From Metal- to Supramolecular Organic Catalysis. Chem. Rec..

[31] Liu K., Shi W., Cheng P. (2011). The coordination chemistry of Zn(II), Cd(II) and Hg(II) complexes with 1,2,4-triazole derivatives. Dalton Trans..

[32] Armanasco N.L., Baker M.V., North M.R., Skelton B.W., White A.H. (1998). Comparative investigation of the Group 6 (Cr, Mo or W) metal carbonyl complexes of 1,3,5-triazacyclohexanes. J. Chem. Soc. Dalton Trans..

[33] Pérez J., Riera L. (2008). Organometallic complexes as anion hosts. Chem. Commun..

[34] Ardizzoia G.A., Brenna S., LaMonica G., Maspero A., Masciocchi N. (2002). Alkyne oligomerization catalyzed by molybdenum(0) complexes. J. Organomet. Chem..

[35] Haasnoot J.G., Vos G., Groeneveld W.L. (1977). 1,2,4-Triazole Complexes, III. Complexes of Transition Metal(II) Nitrates and Fluoroborates. Z. Naturforsch. B.

[36] Haasnoot J.G. (2000). Mononuclear, oligonuclear and polynuclear metal coordination compounds with 1,2,4-triazole derivatives as ligands. Coord. Chem. Rev..

[37] Coelho A.C., Nolasco M., Balula S.S., Antunes M.M., Pereira C.C.L., Paz F.A.A., Valente A.A., Pillinger M., Ribeiro-Claro P., Klinowski J. (2011). Chemistry and Catalytic Activity of Molybdenum(VI)-Pyrazolylpyridine Complexes in Olefin Epoxidation. Crystal Structures of Monomeric Dioxo, Dioxo-μ-oxo, and Oxodiperoxo Derivatives. Inorg. Chem..

[38] Amarante T.R., Neves P., Valente A.A., Paz F.A.A., Fitch A.N., Pillinger M., Gonçalves I.S. (2013). Hydrothermal Synthesis, Crystal Structure, and Catalytic Potential of a One-Dimensional Molybdenum Oxide/Bipyridinedicarboxylate Hybrid. Inorg. Chem..

[39] Zapf P.J., Haushalter R.C., Zubieta J. (1997). Hydrothermal Synthesis and Structural Characterization of a Series of One-Dimensional Organic/Inorganic Hybrid Materials of the [(MoO_3_)_n_(2,2’-bipy)_m_] Family: [MoO_3_(2,2’-bipy)], [Mo_2_O_6_(2,2’-bipy)], and [Mo_3_O_9_(2,2’-bipy)_2_]. Chem. Mater..

[40] Gomes A.C., Neves P., Figueiredo S., Fernandes J.A., Valente A.A., Paz F.A.A., Pillinger M., Lopes A.D., Gonçalves I.S. (2013). Tris(pyrazolyl)methane molybdenum tricarbonyl complexes as catalyst precursors for olefin epoxidation. J. Mol. Catal. A Chem..

[41] Neves P., Nogueira L.S., Gomes A.C., Oliveira T.S.M., Lopes A.D., Valente A.A., Gonçalves I.S., Pillinger M. (2017). Chemistry and Catalytic Performance of Pyridyl-Benzimidazole Oxidomolybdenum(VI) Compounds in (Bio)Olefin Epoxidation. Eur. J. Inorg. Chem..

[42] Lysenko A.B., Senchyk G.A., Domasevitch K.V., Hauser J., Fuhrmann D., Kobalz M., Krautscheid H., Neves P., Valente A.A., Gonçalves I.S. (2015). Synthesis and Structural Elucidation of Triazolylmolybdenum(VI) Oxide Hybrids and Their Behavior as Oxidation Catalysts. Inorg. Chem..

[43] Veiros L.F., Prazeres Â., Costa P.J., Romão C.C., Kühn F.E., Calhorda M.J. (2006). Olefin epoxidation with *tert*-butyl hydroperoxide catalyzed by MoO_2_X_2_L complexes: A DFT mechanistic study. Dalton Trans..

[44] Al-Ajlouni A., Valente A.A., Nunes C.D., Pillinger M., Santos A.M., Zhao J., Romão C.C., Gonçalves I.S., Kühn F.E. (2005). Kinetics of Cyclooctene Epoxidation with *tert*-Butyl Hydroperoxide in the Presence of [MoO_2_X_2_L]-Type Catalysts (L = Bidentate Lewis Base). Eur. J. Inorg. Chem..

[45] Kühn F.E., Groarke M., Bencze É., Herdtweck E., Prazeres A., Santos A.M., Calhorda M.J., Romão C.C., Gonçalves I.S., Lopes A.D. (2002). Octahedral Bipyridine and Bipyrimidine Dioxomolybdenum(VI) Complexes: Characterization, Application in Catalytic Epoxidation, and Density Functional Mechanistic Study. Chem. Eur. J..

[46] Amarante T.R., Neves P., Paz F.A.A., Pillinger M., Valente A.A., Gonçalves I.S. (2012). A dinuclear oxomolybdenum(VI) complex, [Mo_2_O_6_(4,4′-di-*tert*-butyl-2,2′-bipyridine)_2_], displaying the {MoO_2_(*μ*-O)_2_MoO_2_}_0_ core, and its use as a catalyst in olefin epoxidation. Inorg. Chem. Commun..

[47] Lunazzi L., Parisi F., Macciantelli D. (1984). Conformational Studies by Dynamic Nuclear Magnetic Resonance Spectroscopy. Part 27. Kinetics and Mechanism of Annular Tautomerism in Isomeric Triazoles. J. Chem. Soc. Perkin Trans. II.

[48] Bellagamba M., Bencivenni L., Gontrani L., Guidoni L., Sadun C. (2013). Tautomerism in liquid 1,2,3-triazole: A combined energy-dispersive X-ray diffraction, molecular dynamics, and FTIR study. Struct. Chem..

[49] Kottke T., Stalke D. (1993). Crystal handling at low temperatures. J. Appl. Crystallogr..

[50] (2012). SAINT+ Data Integration Engine v. 8.27b© 1997–2012.

[51] Krause L., Herbst-Irmer R., Sheldrick G.M., Stalke D. (2015). Comparison of silver and molybdenum microfocus X-ray sources for single-crystal structure determination. J. Appl. Crystallogr..

[52] Sheldrick G.M. (2014). SHELXT-2014, Program for Crystal Structure Solution.

[53] Sheldrick G.M. (2015). Crystal structure refinement with SHELXL. Acta Crystallogr. Sect. C Struct. Chem..

[54] Sheldrick G.M. (2014). SHELXL Version 2014, Program for Crystal Structure Refinement.

[55] Sheldrick G.M. (2008). A short history of SHELX. Acta Crystallogr. Sect. A Found. Adv..

[56] Hübschle C.B., Sheldrick G.M., Dittric B. (2011). ShelXle: A Qt graphical user interface for SHELXL. J. Appl. Crystallogr..

[57] Brandenburg K. DIAMOND, Version 3.2f.

