# Supplementary material for: A Comparative Study of Molybdenum Carbonyl and Oxomolybdenum Derivatives Bearing 1,2,3-Triazole or 1,2,4-Triazole in Catalytic Olefin Epoxidation
Source: Molecules. 2018 Dec 28;24(1):105. doi: 10.3390/molecules24010105 (PMC6337555; doi:10.3390/molecules24010105)
Supplement: Supplementary file 1 [file molecules-24-00105-s001.zip › ESI-399010-Revised.pdf]

Supporting Information

Article for *Molecules*

A comparative study of molybdenum carbonyl and oxomolybdenum derivatives bearing 1,2,3-triazole or 1,2,4-triazole in catalytic olefin epoxidation

Lucie S. Nogueira, Patrícia Neves, Ana C. Gomes, Tatiana A. Amarante, Filipe A. Almeida Paz, Anabela A. Valente *, Isabel S. Gonçalves, Martyn Pillinger *

Department of Chemistry, CICECO – Aveiro Institute of Materials, University of Aveiro, Campus Universitário de Santiago, 3810-193 Aveiro, Portugal; lucienogueira@ua.pt (L.S.N.); pneves@ua.pt (P.N.); agomes1@ua.pt (A.C.G.); tatiana.amarante@ua.pt (T.A.A.); filipe.paz@ua.pt (F.A.A.P.); atav@ua.pt (A.A.V.); igoncalves@ua.pt (I.S.G.); mpillinger@ua.pt (M.P.)

* Correspondence: atav@ua.pt (A.A.V.), mpillinger@ua.pt (M.P.); Tel.: +351-234-370603

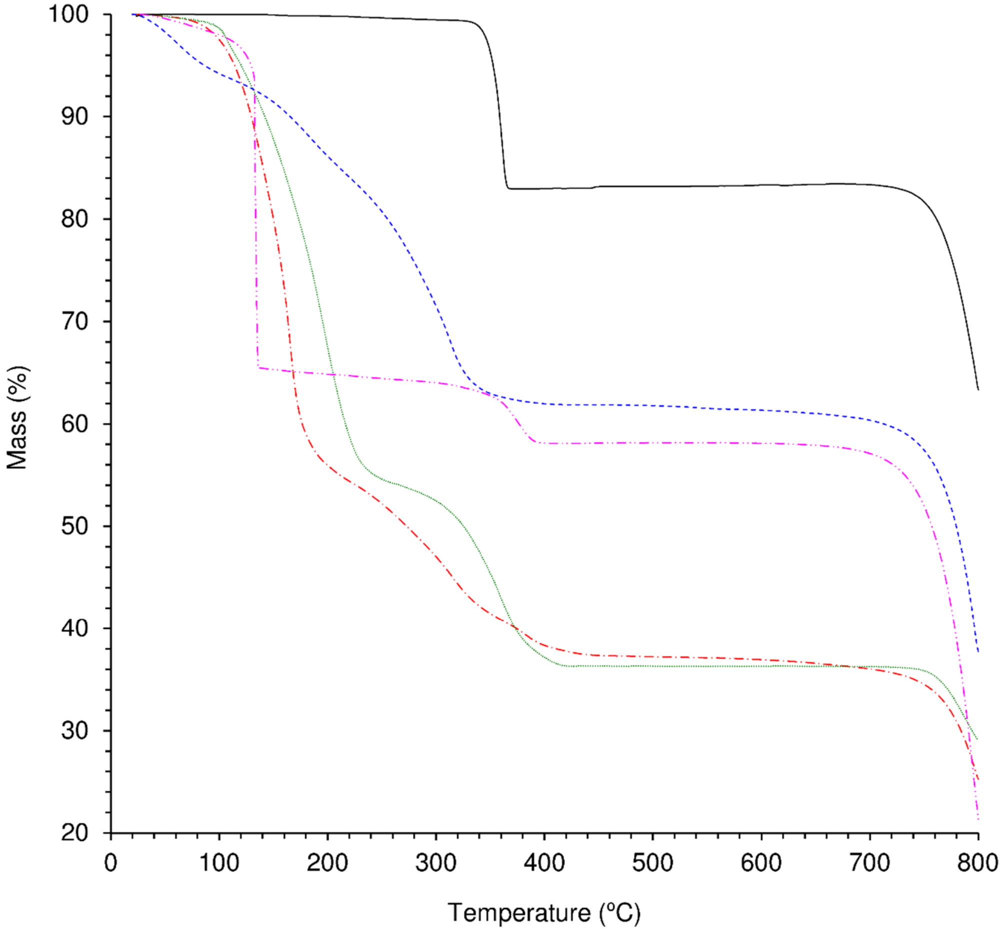

Figure S1. TGA curves for $[Mo(CO)_3(1,2,3\text{-trz})_3]$ (**1**) (· – · – ·), $[Mo(CO)_3(1,2,4\text{-trz})_3]$ (**2**) (·······), $\mathbf{1}^{OD}$ (– – – –), $[MoO_3(1,2,3\text{-trz})_{0.5}]$ (**3**) (——), and $(NH_4)_{1.8}(H_3O)_{0.2}[Mo_2O_2(\mu_2\text{-}O)(O_2)_4(1,2,4\text{-trz})]\cdot H_2O$ (**5**) (· – · · –).

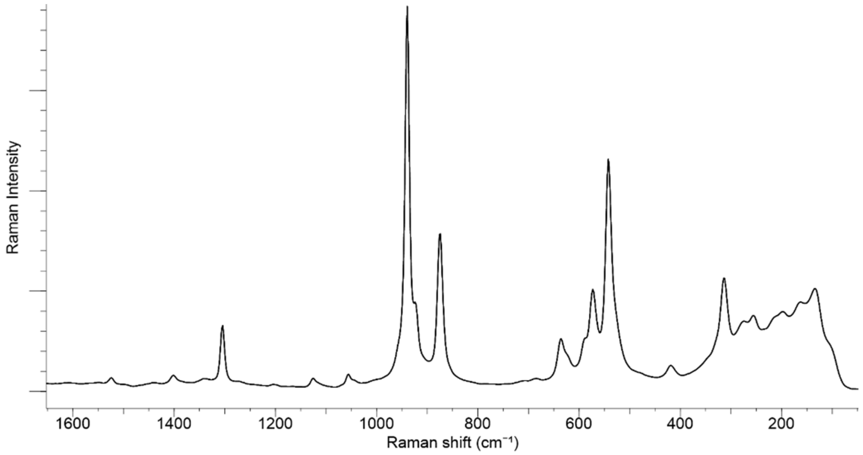

Figure S2. Raman spectrum in the range of 100-1600 cm^{-1} of $(NH_4)_{1.8}(H_3O)_{0.2}[Mo_2O_2(\mu_2\text{-}O)(O_2)_4(1,2,4\text{-trz})]\cdot H_2O$ (**5**).

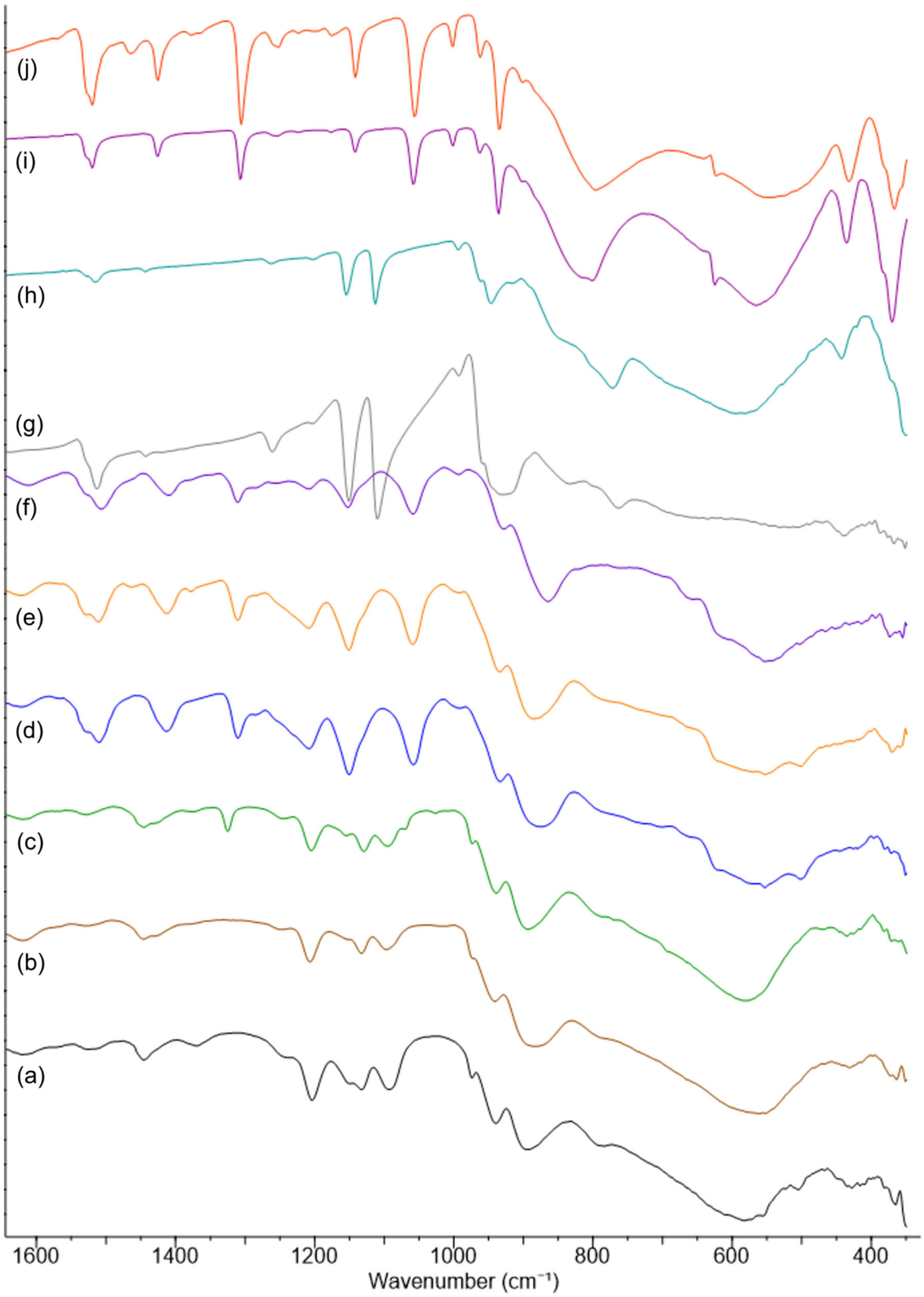

Figure S3. ATR FT-IR spectra in the range of 350-1600 cm^{-1} of (a) $\mathbf{1}^{OD}$, (b) recovered solid $\mathbf{1}^{ODr}$, (c) recovered solid $\mathbf{1}^{r}$, (d) $\mathbf{2}^{OD}$, (e) recovered solid $\mathbf{2}^{ODr}$, (f) recovered solid $\mathbf{2}^{r}$, (g) hybrid **3**, (h) recovered solid $\mathbf{3}^{r}$, (i) hybrid **4**, and (j) recovered solid $\mathbf{4}^{r}$.

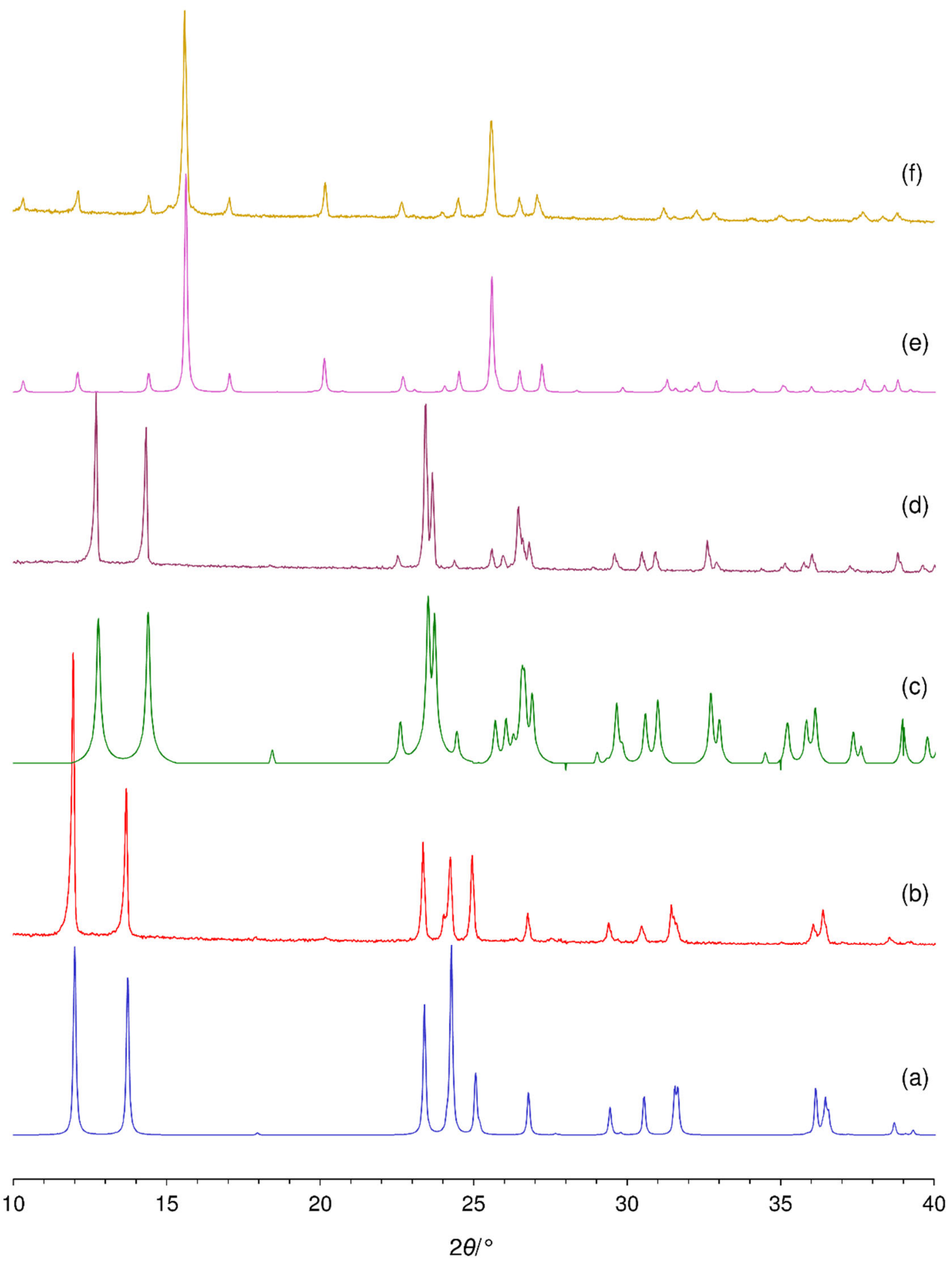

Figure S4. Computed (a,c,e) and experimental (b,d,f) PXRD patterns of (a,b) $[MoO_3(1,2,3\text{-trz})_{0.5}]$ (**3**), (c,d) $[MoO_3(1,2,4\text{-trz})_{0.5}]$ (**4**), and (e,f) $(NH_4)_{1.8}(H_3O)_{0.2}[Mo_2O_2(\mu_2\text{-}O)(O_2)_4(1,2,4\text{-trz})]\cdot H_2O$ (**5**). The program Mercury (copyright CCDC, ver. 3.9) [S1] was used to generate the computed patterns from the crystal structure data published for **3** [S2] and **4** [S3], and the data reported in this paper for **5**.

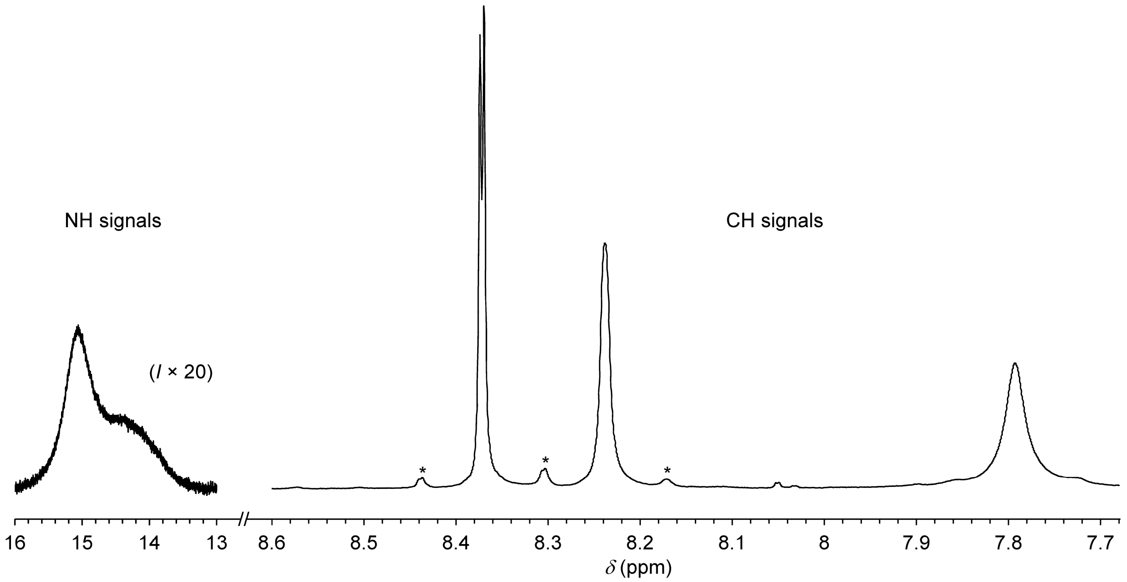

Figure S5. Regions of the ^{1}H NMR spectrum of **1** in $(CD_3)_2CO$ containing signals due to 1,2,3-trz NH and CH protons. The asterisks denote spinning sidebands.

Table S1. Bond distances (Å) and angles (°) for the crystallographically independent Mo^{VI} center present in $(NH_4)_{1.8}|(H_3O)_{0.2}[Mo_2O_2(\mu_2\text{-}O)(O_2)_4(1,2,4\text{-trz})]\cdot H_2O$ (**5**).

Mo1–O1	1.944(3)	O1–Mo1–O3	86.9(4)
Mo1–O2	1.700(6)	O1–Mo1–O6	88.3(3)
Mo1–O3	1.951(6)	O1–Mo1–N1	80.0(3)
Mo1–O4	1.933(7)	O2–Mo1–O1	98.1(3)
Mo1–O5	1.941(6)	O2–Mo1–O3	101.5(3)
Mo1–O6	1.958(6)	O2–Mo1–O4	101.1(3)
Mo1–N1	2.398(9)	O2–Mo1–O5	100.5(3)
		O2–Mo1–O6	101.2(3)
		O2–Mo1–N1	178.0(3)
		O3–Mo1–O6	157.3(3)
		O3–Mo1–N1	78.8(3)
		O4–Mo1–O1	130.2(4)
		O4–Mo1–O3	44.4(3)
		O4–Mo1–O5	89.2(3)
		O4–Mo1–O6	131.1(3)
		O4–Mo1–N1	80.4(3)
		O5–Mo1–O1	131.4(4)
		O5–Mo1–O3	131.6(3)
		O5–Mo1–O6	44.1(3)
		O5–Mo1–N1	80.7(3)
		O6–Mo1–N1	78.6(3)

References

[S1] C. F. Macrae, I. J. Bruno, J. A. Chisholm, P. R. Edgington, P. McCabe, E. Pidcock, L. Rodriguez-Monge, R. Taylor, J. van de Streek, P. A. Wood, *J. Appl. Crystallogr.* **2008**, *41*, 466-470.

[S2] T. R. Amarante, P. Neves, A. A. Valente, F. A. A. Paz, M. Pillinger, I. S. Gonçalves, *J. Catal.* **2016**, *340*, 354-367.

[S3] J. Chuang, W. Oullette, J. Zubieta, *Inorg. Chim. Acta* **2008**, *361*, 2357-2364.
